# Short-Term Side Effects of Low Dose Valproate Monotherapy in Epileptic Children: A Prospective Study

**Published:** 2019

**Authors:** Parisa Nasr ESFAHANI, Jafar NASIRI, Shervin BADIHIAN, Omid YAGHINI

**Affiliations:** 1 Students’ Research Center, School of Medicine, Isfahan University of Medical Sciences, Isfahan, Iran; 2 Child Growth and Development Research Center, Research Institute for Primordial Prevention of Non-communicable Disease, Isfahan University of Medical Sciences, Isfahan, Iran

**Keywords:** Adverse drug reactions, Epilepsy, Pediatrics, Valproate monotherapy

## Abstract

**Objectives:**

Considering the common use of valproate among children, we investigated the short-term side-effects of low dose valproate monotherapy in epileptic children.

**Materials & Methods:**

In this prospective study, 209 epileptic children (48.3% male, mean age: 7.02 ± 3.13 yr) on low therapeutic dose of valproate monotherapy (20-30 mg/kg/d) were enrolled during 2014-2015 in Isfahan Pediatric Neurology Clinic, Isfahan University of Medical Sciences, Isfahan, Iran and side-effects were evaluated through frequent clinical visits and laboratory tests during 6 months of valproate therapy.

**Results:**

Weight gain was reported in 53.1% of patients. Decreased appetite was seen in 11% of patients, more frequent in younger cases (*P*=0.006). Abdominal pain, nausea/vomiting, diarrhea, and constipation were reported in 16.3%, 2.4%, 1.4%, and 1% of patients, respectively. Headache, tremor, dizziness, abnormal color vision, myoclonus, and bruxism were seen in 5.7%, 1.4%, 1%, 1%, 1%, and 0.5% of patients, respectively. Enuresis, hair loss, and skin rash were reported in 8.1%, 6.7%, and 0.5% of patients, respectively. Thrombocytopenia, impaired liver function tests, and leukopenia occurred in 1%, 1%, and 0.5% of patients, respectively.

**Conclusion:**

Low dose valproate monotherapy may cause numerous side-effects, mostly not life-threatening and requiring no action. Besides more reported complications, we observed decreased appetite (among younger patients), enuresis, and abnormal color vision which are onlybriefly discussed in the literature and need to be addressed more.

## Introduction

Valproate (VPA) is a broad-spectrum antiepileptic drug, used for treatment of certain types of seizures since 1970 (1). It can also be used in other conditions including some psychiatric disorders and prophylaxis of migraine (1). Epilepsy is a common disease among children and adolescents. Approximately 10.5 million children under 15 yr (about 0.8%) suffer from active epilepsy, of whom more than 80% live in developing countries (2). Although VPA is an old antiepileptic drug, it is still widely administered for epileptic patients, since it is favorably safe and inexpensive (3).

VPA may cause transient and non-hazardous side effects as well as serious and life-threatening side effects. Transient and non-hazardous side effects include weight gain, drowsiness, transient hair loss, tremor, increased gamma-glutamyl transferase, nausea, headache, and other complications (1, 4). Serious side effects include hepatotoxicity, encephalopathy, coagulation disorders, pancreatitis, and bone marrow suppression (1, 5, 6). Complications of VPA are also classified as gastrointestinal, neurological, metabolic and endocrine, hematologic, pulmonary, renal, dermatologic, mitochondrial, and hepatic adverse events (1). These complications may be associated with factors such as age and dose (1).

Although several studies were conducted on the subject, no study was designed to evaluate these complications in a pediatric population on low dose VPA as monotherapy. We aimed to investigate the short-term complications of low dose VPA in a pediatric population through frequent interviews, clinical visits, and lab tests during a 6-month period of therapy.

## Materials and Methods

This cross-sectional prospective study performed during 2014-2015 in Isfahan Pediatric Neurology Clinic, Isfahan University of Medical Sciences, Isfahan, Iran. Patients ranging from 2 to 15 yr old, with diagnosis of epilepsy supposed to start low therapeutic dose of VPA (20-30 mg/kg/d) as monotherapy were included in the study. The exclusion criteria were defined as any other comorbid disease or medical condition including chronic hepatic disease, chronic renal disease, metabolic syndromes, diabetes, progressive neurological diseases, diseases of digestive system, and coagulation disorders; experiencing worsened seizures after starting VPA; needing higher doses of VPA; taking any other medication affecting body weight. 

The study was approved by the regional bioethics committee of Isfahan University of Medical Sciences and informed consent was obtained from all patients or their parents.

VPA was started for patients with the decision of pediatric neurologists and based on the recent guidelines on treatment of epilepsy (7). After recruitment, a thorough history was taken from patients and they underwent complete physical and neurological examination. We designed a data collection questionnaire which included patients’ characteristics and demographic data, initial weight, seizure type, prescribed dose of VPA, and possible side effects of the drug (based on previous studies), including weight gain, change in appetite (according to parents’ evaluation), abdominal pain, nausea/vomiting, headache, enuresis, hair loss (diffuse reduction of hair defined as losing more than 100 hairs daily, evaluated by parents), diarrhea, constipation, tremor, skin rash, dizziness, abnormal color vision (sensible changes in the color vision or dominance of certain colors stated by the patient), bruxism, and myoclonus. To evaluate weight gain, we weighed patients at two time-points: before taking VPA, and after 6 months of taking VPA. As we expected no considerable changes in the children’s height and in turn, their body mass index, we calculated weight-for-age Z-score for each subject in these two time-points, using national z-score tables for boys and girls. Two amounts were compared to each other then, and increased z-score for ≥1 unit was considered as weight gain (8).

**Table 1:** Complications of valproate and their association with patients’ age

**Category**	**Subcategory**	**Frequency (%)**	**Age (Mean ± SD)**	**P-value**	**Naranjo scale**
**Weight gain**	Yes	111 (53.1)	7.29 ± 3.03	0.121	Probable ADR
	No	98 (46.9)	6.79 ± 3.19		
**Appetite**	Increased	69 (33)	8 ± 3.28	**0.006**	Probable ADR
	Decreased	23 (11)	6.72 ± 2.08		Possible ADR
	No change	117 (56)	6.51 ± 3.10		
**Abdominal pain**	Yes	34 (16.3)	7.75 ± 2.73	0.54	Probable ADR
	No	175 (83.7)	6.88 ± 3.20		
**Nausea/vomiting**	Yes	5 (2.4)	9.20 ± 4.09	0.160	Probable ADR
	No	204 (97.6)	6.97 ± 3.10		
**Headache**	Yes	12 (5.7)	10.50 ± 3.15	**<0.001**	Probable ADR
	No	197 (94.3)	6.81 ± 3.01		
**Enuresis**	Yes	17 (8.1)	7 ± 3.15	0.895	Possible ADR
	No	192 (91.9)	7.03 ± 3.14		
**Hair loss**	Yes	14 (6.7)	8.25 ± 3.31	0.142	Probable ADR
	No	195 (93.3)	6.94 ± 3.11		
**Diarrhea**	Yes	3 (1.4)	6.67 ± 1.53	0.912	Probable ADR
	No	206 (98.6)	7.03 ± 3.15		
**Tremor**	Yes	3 (1.4)	12 ± 2	**0.017**	Probable ADR
	No	206 (98.6)	6.95 ± 3.09		
**Skin rash**	Yes	1 (0.5)	2	**0.029**	Probable ADR
	No	208 (99.5)	7.05 ± 3.12		
**Dizziness**	Yes	2 (1)	7.50 ± 0.71	0.540	Probable ADR
	No	207 (99)	7.02 ± 3.15		
**Constipation**	Yes	2 (1)	5	0.340	Probable ADR
	No	207 (99)	7.04 ± 3.14		
**Bruxism**	Yes	1 (0.5)	7	0.871	Possible ADR
	No	208 (99.5)	7.02 ± 3.14		
**Myoclonus**	Yes	2 (1)	8.50 ± 4.95	0.596	Possible ADR
	No	207 (99)	7.01 ± 3.13		
**Abnormal color vision**	Yes	2 (1)	6.5 ± 2.12	0.981	Possible ADR
	No	207 (99)	7.03 ± 3.15		
**Thrombocytopenia**	Yes	2 (1)	5.5 ± 0.71	0.520	Probable ADR
	No	207 (99)	7.04 ± 3.15		
**Leukopenia**	Yes	1 (0.5)	15	**0.029**	Probable ADR
	No	208 (99.5)	6.99 ± 3.09		
**Increased liver enzymes**	Yes	2 (1)	4.5 ± 0.71	0.199	Probable ADR
	No	207 (99)	7.05 ± 3.14		

SD: standard deviation; ADR: adverse drug reaction

Blood samples were also taken to evaluate complete blood count with differentials (CBC-diff), serum glutamic oxaloacetic transaminase (SGOT), and serum glutamic pyruvic transaminase (SGPT) at four time-points: before starting the medication, 1 month, 3 months, and 6 months after starting the medication. Blood samples were obtained between 8 and 9 in the morning, after an overnight fast and before breakfast. Platelet count <150,000 /µL was considered as thrombocytopenia, WBC <4000 /µL was considered as leukopenia and increased SGOT and SGPT for more than two times were considered as increased liver enzymes. In patients with serious side effects, drug intolerance, and impaired lab tests, VPA was discontinued and was replaced with another drug. All the observed adverse drug reactions were rated using Naranjo scale (9) into one of the categories of highly probable, probable, possible, and doubtful. Naranjo scale determines whether an adverse drug reaction is actually due to the medication or it is caused by other factors. This scale evaluates the probability of a complication through 10 questions (9).

We reported data using descriptive statistics for frequencies and analytical statistics (Mann-Whitney test) for analysis of possible differences, using SPSS 19 (Chicago, IL, USA). A *P*-value less than 0.05 was considered as significant.

## Results

Overall, 229 patients were included initially and 20 of them were excluded (13 patients with worsened seizures and 7 cases who needed higher doses of VPA). Of the remained cases, 101 patients (48.3%) were male and the mean age (standard deviation [SD]) was 7.02 (3.13). Most patients had generalized seizures (75.6%) followed by absence seizures (9.6%), partial seizures (8.6%), and myoclonic seizures (6.2%).

Adverse drug reactions and their association with age are summarized in Table 1. Weight gain was seen in 111 patients (53.1%) while 33% of patients reported increased appetite and 11% of them reported decreased appetite. Older patients experienced increased appetite more frequently (*P*=0.006). Abdominal pain was reported in 16.3% of patients. It was mostly transient and lead to VPA discontinuation in none of the cases.

Twelve patients (5.7%) complained of headache, seen more in older ages (*P*<0.001). Enuresis and hair loss were reported in 17 (8.1%) and 14 (6.7%) patients respectively. Tremor and diarrhea were seen in 3 (1.4%) patients, and tremor was seen more in older ages (*P*=0.017). Only 2 patients (0.1%) presented one of the symptoms of dizziness, constipation, abnormal color vision, and myoclonus and one patient (0.5%) experienced skin rash or bruxism. Abnormal color vision was reversed by drug discontinuation.

Regarding patients’ lab tests, 2 cases (1%) developed thrombocytopenia, one patient (0.5%) had leukopenia, and in 2 patients had (1%) impaired liver function tests. In general, side effects lead to drug discontinuation in 12 patients, including 2 patients with thrombocytopenia, 1 patient with leukopenia, 7 patients with drug intolerance, and 2 patients with abnormal color vision.

Regarding all the side effects (except appetite change), 101 patients (48.3%) experienced one side effect, 29 patients (13.9%) experienced two side effects, 13 patients (6.2%) experienced three side effects and 3 patients (1.5%) experienced four or five side effects. Therefore, only 30.1% of patients experienced no side effects.

## Discussion

Weight gain is one of the common complications of VPA seen in 53.1% of our patients, without any association with age. A study on Iranian children showed weight gain due to VPA in 40% of patients (10), however, another study reported weight gain in 58% of their cases (11). A negative correlation between weight gain and duration of treatment was reported before (12). weight gain is seen more in epileptic patients over 10 yr old (13). In contrast, no association was found between weight gain and age, gender, and drug dose in another study (14), which is also consistent with our findings.

In a clinical trial on epileptic children, increased appetite was reported in all of their subjects with marked weight gain (15). Increased and decreased appetite were seen in 33% and 11% of our patients, respectively. We found that patients with increased appetite are older than those with decreased or unchanged appetite. Based on our results, decreased appetite may be a complication of low dose VPA particularly in younger ages, however, we believe that evaluation of appetite may not be precise enough in our study since it was evaluated by parents. Decreased appetite has not been reported in previous studies and needs to be investigated more in the future, especially in younger patients. 

Other gastrointestinal side effects in our study included abdominal pain (16.3%), nausea/vomiting (2.4%), constipation (1%), and diarrhea (1.4%). Gastrointestinal complications are known as common side effects of VPA occurring in up to 50% of patients (1, 4, 16). These symptoms are usually transient and require no intervention (4). Sudden onset of abdominal pain may be due to acute pancreatitis, which is an uncommon but serious side effect of VPA (1). We had no report of pancreatitis among our cases.

Neurological adverse effects reported among our cases were as follows: Dizziness (1%), headache (5.7%), tremor (1.4%), abnormal color vision (1%), myoclonus (0.5%), and bruxism (0.5%). We found headache more in older patients. Older patients are more capable of expressing their headache and this may be a factor affecting our results. Dizziness and headache are suggested as VPA side effects in previous studies (17). Tremor is commonly reported as a side effect of VPA (17) occurring in 1%-6% of patients (4) and is known to be dose dependent (18). Bruxism and myoclonus are rarely reported in the literature as side effects of VPA (1, 19).

We found enuresis in 8.1% of our patients. Enuresis due to VPA intake is less being addressed in the literature (18). In a study, 72 epileptic patients ranging from 2 to 15 yr were on long-term VPA therapy and reported enuresis in 17 of them (24%), which stopped after discontinuation of the drug. Age was the only factor introduced as the predicting factor for enuresis in this study (18). Moreover, Egger and Brett reported enuresis in 7% of their study group (100 subjects) (20). We confirm previous findings on the frequency of enuresis after VPA therapy, but after a short-term period, however, we found no association between enuresis and age. Enuresis was a new finding in our patients and they did not have history of enuresis due to seizures.

Hair loss was reported by 6.7% of our patients. Several studies have addressed the possible effects of VPA on hair growth, such as alopecia, thinning hair, and color change (4, 21). The occurrence of this condition has been reported from 3.5% to 11% in previous studies (21). Hair loss due to VPA is usually scattered, without any scars, occurring 3 to 6 months after starting VPA, and is suggested to be associated with higher doses of the medication in a few studies (21). Only one case (0.5%) complained of skin rash in our study. Skin rash is a rarely reported complication of VPA, mostly seen in combination therapies and is disappeared after drug discontinuation (4, 22).

VPA might impair color perception significantly (23). A study on adolescents on VPA monotherapy showed that VPA can significantly affect central and para-central color vision after a short-term period (24). Ophthalmologic examination of these patients is recommended in case ophthalmic symptoms develop (24). Two of our patients (1%) complained of abnormal color vision during their treatment. The complication was reversed by VPA discontinuation. Our patients described their condition as seeing objects with abnormal colors or dominancy of certain colors. Considering the reversibility of these symptoms, it seems not to be a serious complication of VPA.

We found thrombocytopenia in 1% of our patients. Thrombocytopenia is a dose-dependent complication of VPA (1, 4). This condition has been reported in a wide range of 1% to 32% in patients with different age groups and is known as the most common hematologic side effect of VPA (1, 18, 25, 26). Thrombocytopenia is reported more frequently due to multiple drug treatment, being female, and initial thrombocytopenia (27). Children are more prone to thrombocytopenia because of using higher doses of the drug compared to adults. Reduction or discontinuation of VPA results in increasing platelet counts in the next few days in these patients (25).

One of our patients (0.5%) developed leukopenia during the study. Leukopenia is rarely reported as a complication of VPA and is reversible by drug discontinuation and happens more in combination therapies (28). Bone marrow suppression and production of antibodies against thrombocytes may be responsible for decrease in blood cell lines (1). Increased liver enzymes were observed in 1% of our patients. Elevation of serum transaminases is a common and mainly reversible side effect of VPA, although it may develop to more severe and irreversible conditions such as severe hepatotoxicity (1, 4, 5, 29). Hepatotoxicity is reported to happen more in younger ages (30) and higher administered doses of VPA (29).

In this study, we had some limitations that should be noted. First, this study was open and uncontrolled. Therefore, we could not compare the frequency of complications with the normal population which makes it hard to distinguish adverse events from adverse effects. Second, some complications, such as hair loss and color vision, were evaluated based on parents’ or patients’ evaluation, while measuring them with more objective tools (for example dermatologic or ophthalmologic examination) might change the results. These results may be underestimated or overestimated in our study, based on parents’ precision and their obsession with their children. Third, we studied subjects who were on low dose VPA monotherapy, while VPA may develop more side effects on polytherapy and higher doses. Forth, there are some other side effects of VPA including oligomenorrhea in female adolescents and decreased serum levels of vitamin D not measured in this study. Despite these limitations, this is the first study that evaluates side effects of low dose VPA monotherapy in epileptic children in a prospective design.

**In conclusion,** low dose VPA monotherapy may cause numerous side effects, however, most of them are not dangerous and life-threatening. We reported decreased appetite especially in patients with younger ages which needs to be investigated more in future studies. Moreover, we addressed enuresis and abnormal color vision briefly discussed in the literature and need to be studied more. Further studies with larger study population and longer follow up period are recommended to evaluate our findings.
